# Specificity of 2 peripartum blood markers for early-lactation acute uterine inflammation in pasture-fed, seasonal-calving dairy cows

**DOI:** 10.3168/jdsc.2023-0509

**Published:** 2024-04-20

**Authors:** O.K. Spaans, J.R. Roche, C.R. Burke, C.V.C. Phyn, S. McDougall, C.B. Reed, B. Kuhn-Sherlock, A. Hickey, A. Heiser, M.A. Crookenden

**Affiliations:** 1 DairyNZ Limited, Hamilton, 3240, New Zealand; 2 School of Biological Sciences, University of Auckland, Auckland, 1142, New Zealand; 3 Cognosco, AnexaFVC, Morrinsville, 3300, New Zealand; 4 AgResearch, Hopkirk Research Institute, Grasslands Research Centre, Palmerston North, 4410, New Zealand

## Abstract

A delayed recovery of the reproductive tract from natural inflammatory processes associated with postpartum involution will compromise further reproductive function. Following a literature review, we selected serum amyloid A (SAA) and α1-acid glycoprotein (α1-AGP) to assess as potential circulating markers of acute uterine inflammation, as concentrations of these 2 acute phase proteins were reported to be elevated early postpartum in dairy cows with active uterine infection. Convenience serum samples from an induced model of uterine infection were used to measure concentrations of these markers. Infection was induced by infusing either 10^7^ or 10^9^ cfu of *Trueperella pyogenes* (n = 9 cows each; bacteria group n = 18) or saline as a control (n = 18) into the uterus at 48 d postpartum. Although infection stimulated an increase in uterine polymorphonuclear neutrophils, SAA and α1-AGP concentrations in serum were not different between infusion groups. Cows were subsequently classified into uterine health groups based on the presence of endometritis, with or without the presence of *T. pyogenes* in uterine culture in response to uterine infusion. Mean SAA concentrations were greater in cows that were either endometritis negative–infection positive (n = 9), endometritis positive–infection negative (n = 5), or endometritis positive–infection positive, compared with the endometritis negative–infection negative (n = 11) cows. There was no difference between uterine health groups for α1-AGP concentrations.

## Highlights

• Circulating inflammatory markers increase in dairy cows following calving.
• Selected markers were measured in a uterine infection model at 7 weeks postpartum.
• Inflammation was confirmed by elevated uterine polymorphonuclear neutrophils.
• Serum amyloid A also increased with uterine infection and inflammation.
• No difference was observed between groups for alpha-1-acid glycoprotein.

## Graphical Abstract

**Figure:**
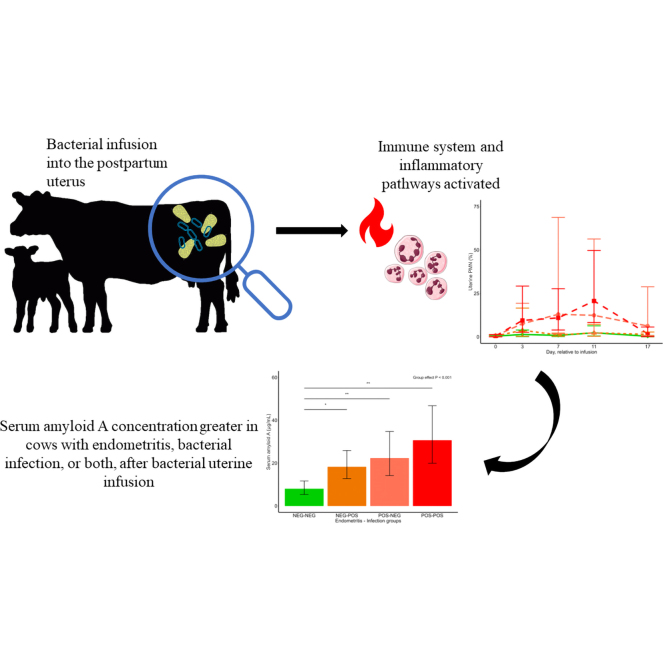
**Summary:** A common pathogen to the postpartum uterine environment was infused into the uterus of dairy cows to induce a uterine infection at 7 weeks postpartum. A control group received a uterine infusion of saline. As a result, inflammatory pathways were activated, characterized by the increase of uterine polymorphonuclear neutrophils for 2 weeks following the infusion. We selected circulating markers of inflammation, serum amyloid A and alpha-1-acid glycoprotein, to measure in the bacterial and saline infusion groups and identify whether these markers were specific for uterine inflammation. These markers of inflammation were reported in other studies to be elevated early postpartum in dairy cows with a uterine infection. Concentrations of serum amyloid A were elevated in cows that had a uterine infection, inflammation, or both, compared with cows that had no infection or inflammation. There was no difference between groups for alpha-1-acid glycoprotein concentration.

In pasture-based, seasonal calving systems, reproductive efficiency is essential to maintain the 365-d intercalving interval needed to synchronize feed demand with pasture supply and maximize profitability (Roche et al., 2017). Although compromised reproductive function is multifactorial and complex, postpartum uterine infection and inflammation are widely regarded as important elements (LeBlanc, 2020). Bacteria can be isolated from the uterus in over 90% of cows in the early postpartum period (Sheldon et al., 2009). Bacterial exposure at calving stimulates the immune response and associated pro-inflammatory processes as part of the acute phase response (**APR**) to eliminate foreign entities and minimize the risk of infection (Sheldon et al., 2009). Furthermore, the bacterial exposure coincides with a period of peripartum immune dysregulation, and includes changes in neutrophil function, such as reduced neutrophil adhesion and myeloperoxidase activity (Crookenden et al., 2016, 2019). Dysregulation of immune, inflammatory, and metabolic pathways during the periparturient period have been implicated in the pathogenesis of postpartum diseases, including reproductive tract conditions such as endometritis (Huzzey et al., 2009; Bogado Pascottini and LeBlanc, 2020). Despite the necessity for and naturalness of the peripartum inflammatory processes, timely resolution is critical; a failure to resolve either infection or the inflammatory response associated with the infection negatively affects reproductive outcomes (Williams et al., 2005; Dubuc et al., 2010; Bradford et al., 2015).

Based on a review of the literature, we proposed that serum amyloid A (**SAA**) and α1-acid glycoprotein (**α1-AGP**) be investigated for their specificity to uterine inflammation, as both markers have been associated with reproductive tract inflammation and bacterial presence. For example, increased concentrations of α1-AGP were associated with uterine *Escherichia coli* and *Trueperella pyogenes*, and a high uterine bacterial growth score in the weeks following calving (Sheldon et al., 2001; Williams et al., 2005, 2007). We hypothesized that circulating concentrations of SAA and α1-AGP would be greater in cows with an induced uterine infection of *T. pyogenes* at 48 d postpartum.

Recognizing the increasing evidence that the inflammatory response to trauma or infection can present as various uterine disorders that may occur independently, cows were subsequently categorized into uterine health groups. Uterine PMN percentage and presence of *T. pyogenes* in culture from 3 to 17 d postinfusion were used to classify cows, regardless of bacterial infusion treatment, into these groups and compare profiles of circulating markers SAA and α1-AGP. A better understanding of the profile of inflammatory markers in response to uterine trauma or infection may help identify the pathways involved in the failure to resolve inflammation. Therefore, we aimed to identify whether the profile of circulating concentrations of 2 inflammatory markers were similar between cows that had (1) no endometritis or bacterial infection, (2) had endometritis in the absence of *T. pyogenes* in uterine bacterial culture, (3) did not develop endometritis but had *T. pyogenes* isolated in culture, and (4) both endometritis and *T. pyogenes* were isolated.

Serum samples used in the current study were a convenience sample from a previously completed “parent” experiment (McDougall et al., 2022). The study protocol was not registered, and study data are not publicly available.

The design and sampling protocols for the parent experiment are outlined in McDougall et al. (2022). Briefly, dairy cows (n = 36) of mixed age, predominantly Holstein-Friesian breed (i.e., ≥12/16 Holstein-Friesian), and 48 ± 4.2 DIM were enrolled, blocked by age, and randomly allocated to one of 3 uterine infusion treatment groups (random numbers generated in Microsoft Excel): saline (**SAL**; n = 18), 10^7^ (n = 9), or 10^9^ (n = 9) cfu *T. pyogenes* as part of a negative-controlled intervention study to develop an intrauterine infection model in postpartum dairy cows (McDougall et al., 2022). In the current study, due to no significant differences in SAA or α1-AGP concentration, or PMN percentage (*P* = 0.40, 0.72, and 0.65, respectively) between the 10^7^ and 10^9^ cfu infusion groups (mean and SD of the PMN percentage was 13 ± 18.9% and 14 ± 22.2% for 10^7^ and 10^9^ cfu infusion groups, respectively), they were combined into one treatment group (bacteria group; **BACT**; n = 18). For the SAL and BACT groups, respectively: BCS was 3.8 ± 0.22 (mean ± SD) and 3.8 ± 0.17 (1–10 point scale; (Roche et al., 2004); BW was 418 ± 50.9 kg and 417 ± 47.0 kg for the 3 wk following infusion; and parity was 2 ± 1.0 and 2 ± 1.5.

Blood samples were collected immediately before infusion on d 0 (48 ± 4.2 DIM), and then at d 3, d 7, d 11 ± 4.1 (d 9 to 12), and d 17 ± 4.3 (d 15 to 18) postinfusion. Five cows (n = 2, SAL; n = 3, BACT) did not have a d 17 postinfusion sample collected. Cows with missing samples were included in the analyses without these data being available. Blood (10 mL) was collected in duplicate from the coccygeal (tail) vein into plain evacuated blood tubes (BD Bioscience). Blood samples were centrifuged at 1,200 × *g* for 12 min at 4°C and the serum aspirated and stored at −20°C until protein analysis.

Cells from the endometrium were collected immediately following the blood samples on d 0, 3, 7, 11, and 17 postinfusion using a cytobrush (Pap endocervical sample brush; Ebos Group Ltd., Christchurch, New Zealand), and PMN counted as previously outlined in McDougall et al. (2022). A second cytobrush sample was placed in Amies transport medium (Copan, Fort Richard Laboratories, Auckland, New Zealand) for microbial culture of *T. pyogenes*, the methods for which are outlined in McDougall et al. (2022).

Proteins α1-AGP (Life Diagnostics, West Chester, PA) and SAA (TriDelta Development Ltd., Maynooth, Co. Kildare, Ireland) were measured in serum from cows in the SAL and BACT groups at d 0, 3, 7, 11, and 17 postinfusion. Dilution of serum samples with assay buffer was optimized to 1:150 for SAA and 1:20,000 for α1-AGP using a 3-step dilution series. Intra-assay average CV were 10.9% and 4.7% for SAA and α1-AGP, respectively, and inter-assay CV were 12.1% and 12% for SAA and α1-AGP, respectively, with a lower limit of quantification of 1.5 µg/mL and 7 ng/mL, respectively.

Data were log_10_ transformed to achieve homogeneity of variance for serum SAA, α1-AGP, and uterine PMN and analyzed in SAS (SAS Studio 3.8, SAS release 9.04). The experimental unit was the cow; no cows or data points were excluded from the analyses. Parity was categorical (primiparous, parity 2–3, and 4+) in the models. A repeated measures mixed model (PROC MIXED) was undertaken to compare mean serum SAA and α1-AGP concentrations, and uterine PMN percentage between the *T. pyogenes* 10^7^ and 10^9^ cfu infusion groups. A repeated measures mixed model with backward stepwise elimination was undertaken to determine the effect of day, infusion group, and their interaction on concentrations of serum SAA and α1-AGP, and uterine PMN percentage. The d 0 concentration of SAA and α1-AGP, and the d 0 uterine PMN percentage were included in the respective models as a fixed effect and retained in the final model regardless of significance. Fixed effects (parity and calving season day; number of days from June 1 to the actual calving date) were retained in the model if they were significant (*P* < 0.05). A separate autoregressive covariance structure for infusion group was chosen. Effects retained in the final model for SAA were d 0 concentration (*P* < 0.001), infusion group (*P* = 0.27), day (*P* < 0.001), and infusion group by day (*P* = 0.18). For uterine PMN percentage, effects retained were d 0 uterine PMN (*P* = 0.31), parity group (*P* < 0.001), infusion group (*P* < 0.001), day (*P* = 0.0082), and infusion group by day (*P* = 0.18). Fixed effects retained in the final model for α1-AGP were d 0 concentration (*P* < 0.001), infusion group (*P* = 0.73), day (*P* = 0.036), and infusion group by day (*P* = 0.68).

Cows were subsequently classified into 4 uterine health groups based on uterine PMN and bacterial infection of *T. pyogenes*. For uterine PMN, cows were classified as endometritis positive (**POS**) if there were ≥5% PMN at 2 or more time points, and otherwise classified as endometritis negative (**NEG**). For bacterial infection, cows were classified into infection positive if there were >3 colonies of *T. pyogenes* in the microbial culture at 2 or more time points, and otherwise infection negative. These were further combined into endometritis negative–infection negative (**NEG–NEG**; n = 11), endometritis negative–infection positive (**NEG–POS**; n = 9), endometritis positive–infection negative (**POS–NEG**; n = 5), and endometritis positive–infection positive (**POS–POS**; n = 11) groups.

A repeated measures mixed model with backward stepwise elimination was undertaken to determine the effect of day, uterine health group, and their interaction on concentrations of serum SAA and α1-AGP. The d 0 concentration of SAA and α1-AGP, and the infusion group (i.e., SAL or BACT) were used as fixed effect covariates for the respective models and retained in the final model regardless of significance. Fixed effects (parity and calving season day) were retained in the model if they were significant (*P* < 0.05). A separate autoregressive covariance structure for uterine health group was chosen. Effects retained in the final models for SAA were d 0 concentration (*P* = 0.051), infusion group (*P* = 0.13), day (*P* < 0.001), uterine health group (*P* < 0.001), and uterine health group by day (*P* = 0.95). For α1-AGP, fixed effects retained were parity group (*P* = 0.050), d 0 concentration (*P* < 0.001), infusion group (*P* = 0.64), day (*P* = 0.015), uterine health group (*P* = 0.11), and uterine health group by day (*P* = 0.78). For each model, the Tukey-Kramer method for multiple comparisons adjustment was applied. Least squares means and the 95% upper and lower confidence limits were back-transformed by raising 10 to the respective values. The back-transformed LSM (i.e., geometric mean) and the 95% CI are presented in figures, tables, and text.

The first analysis involved comparing uterine PMN percentage and concentrations of circulating markers in saline and bacterial infusion groups. Following infusion, uterine PMN in the BACT group was greater than the SAL group (Table 1; *P* < 0.001). Uterine PMN initially increased with days postinfusion, from a mean of 3.7% [CI: 2.12–5.98%] at d 3 postinfusion to 5.2% [3.14–8.36%] at d 11 postinfusion, then decreased to 1.3% [0.53–2.60%] at d 17 postinfusion (Table 1; *P* < 0.01). There was no effect of infusion group, or an infusion group by day interaction; however, there was an effect of day postinfusion on circulating concentrations of both SAA and α1-AGP (Table 1; *P* < 0.05). Concentrations of SAA were greatest at d 3 postinfusion, at 58.7 µg/mL [41.63–82.54 µg/mL], decreasing to 15.8 µg/mL [11.03–22.57 µg/mL] at d 7 postinfusion, to 6.5 µg/mL [11.03–22.57 µg/mL] at d 11 postinfusion, and increasing to 13.3 µg/mL [8.91–19.52 µg/mL] at d 17 postinfusion. The profile of serum concentrations of α1-AGP was slightly different, increasing from 0.72 mg/mL [0.606–0.834 mg/mL] at d 3 postinfusion to 0.95 [0.823–1.082 mg/mL] at d 7 postinfusion, and decreasing thereafter to 0.77 mg/mL [0.660–0.895 mg/mL] and 0.73 mg/mL [0.610–0.859 mg/mL] at d 11 and 17 postinfusion, respectively.

**Table 1:** Geometric means and 95% lower (LCI) and upper (UCI) confidence intervals of uterine PMN percentage and concentrations of serum amyloid A and α1-acid glycoprotein for cows in the saline (SAL; n = 18) and bacteria (BACT; n = 18) infusion groups from d 3 to 17 postinfusion

Item	SAL			BACT			*P*-value^1^		
	Mean	LCI	UCI	Mean	LCI	UCI	Infuse	Day	Infuse × Day
Uterine PMN (%)							<0.001	<0.01	0.18
d 3	2.2	0.93	4.40	5.7	2.72	11.19			
d 7	1.4	0.46	3.07	5.4	2.53	10.55			
d 11	1.8	0.69	3.74	12.7	6.44	24.15			
d 17	1.0	0.15	2.39	1.8	0.47	4.32			
Serum amyloid A (μg/mL)							0.27	<0.001	0.18
d 3	46.8	30.28	71.98	73.5	43.18	124.75			
d 7	16.5	10.45	25.71	15.2	8.61	26.37			
d 11	4.3	2.45	7.06	9.6	5.27	16.84			
d 17	15.4	9.46	24.69	11.4	6.00	21.00			
α1-Acid glycoprotein (mg/mL)							0.73	<0.05	0.68
d 3	0.77	0.626	0.917	0.67	0.503	0.851			
d 7	0.90	0.749	1.063	1.00	0.799	1.216			
d 11	0.76	0.619	0.909	0.79	0.612	0.985			
d 17	0.70	0.556	0.854	0.76	0.573	0.976			

1 *P*-values presented for effect of uterine infusion group (Infuse), day postinfection (Day), and the interaction of infusion group and day postinfusion (Infuse × Day) in the repeated measures mixed models.

It was subsequently hypothesized that the presence or absence of endometritis and concurrent presence or absence of *T. pyogenes* may affect the profile of inflammatory markers. When cows were classified into uterine health groups based on uterine PMN percentage (i.e., endometritis positive or negative) and presence of *T. pyogenes* at 2 or more time points postinfusion (i.e., infection positive or negative), there was an effect on the circulating concentrations of SAA. Mean concentrations for NEG–NEG, POS–NEG, NEG–POS, and POS–POS groups were 8.0 µg/mL [5.35–11.65 µg/mL], 18.2 µg/mL [12.73–25.87 µg/mL], 22.3 µg/mL [14.14–34.73 µg/mL], and 30.6 µg/mL [19.90–46.77 µg/mL], respectively (*P* < 0.001; Table 2; Figure 1). There was also an effect of day postinfusion on both SAA and α1-AGP concentrations. With the uterine health group included in the model, SAA and α1-AGP concentrations were similar to models with the bacterial infusion group, with an effect of day postinfusion. Concentrations of SAA were greatest at d 3 postinfusion, with a mean of 62.1 µg/mL [44.11–87.13 µg/mL], decreasing to 16.5 µg/mL [11.51–23.42 µg/mL] at d 7 postinfusion and 6.6 µg/mL [4.46–9.66 µg/mL] at d 11 postinfusion, and increasing to 14.1 µg/mL [9.53–20.53 µg/mL] at d 17 postinfusion (Table 2; *P* < 0.001). At d 3 postinfusion, α1-AGP concentrations were 0.79 mg/mL [0.618–0.898 mg/mL], increasing to 1.04 mg/mL [0.901–1.184 mg/mL] at d 7 postinfusion, and decreasing again to 0.84 mg/mL [0.721–0.977 mg/mL] at d 11 postinfusion and 0.79 mg/mL [0.660–0.927 mg/mL] at d 17 postinfusion (Table 2; *P* < 0.05).

**Table 2:** Geometric means and 95% lower (LCI) and upper (UCI) confidence intervals of concentrations of serum amyloid A and α1-acid glycoprotein from d 3 to 17 postinfusion for cows classified into uterine health groups^1^

Item	NEG–NEG			NEG–POS			POS–NEG			POS–POS			*P*-value^2^		
	Mean	LCI	UCI	Mean	LCI	UCI	Mean	LCI	UCI	Mean	LCI	UCI	Group	Day	Group × Day
Serum amyloid A (μg/mL)													<0.001	<0.001	0.95
d 3	30.3	17.01	53.48	55.2	28.83	104.80	87.9	37.94	201.86	100.0	48.97	203.36			
d 7	7.4	3.80	13.53	19.6	9.96	37.86	18.3	7.47	43.14	27.0	12.84	55.60			
d 11	2.2	0.82	4.51	6.9	3.19	13.85	6.3	2.20	15.65	17.6	8.18	36.52			
d 17	6.8	3.24	13.23	13.9	6.92	27.07	22.4	9.23	52.30	18.0	7.29	42.56			
α1-Acid glycoprotein (mg/mL)													0.11	<0.05	0.77
d 3	0.74	0.539	0.966	0.76	0.542	1.008	0.93	0.699	1.195	0.73	0.475	1.027			
d 7	0.83	0.619	1.070	0.96	0.716	1.234	1.25	0.982	1.561	1.13	0.821	1.501			
d 11	0.69	0.499	0.915	0.69	0.483	0.932	0.97	0.729	1.235	1.05	0.751	1.405			
d 17	0.75	0.530	0.996	0.70	0.492	0.943	0.79	0.576	1.037	0.92	0.590	1.312			

1 NEG–NEG: endometritis negative–infection negative; NEG–POS: endometritis negative–infection positive; POS–NEG: endometritis positive–infection negative; POS–POS: endometritis positive–infection positive.

2 *P*-values presented for effect of uterine health group (Group), day postinfection (Day), and the interaction of uterine health group and day postinfusion (Group × Day) in the repeated measures mixed models.

**Figure 1:**
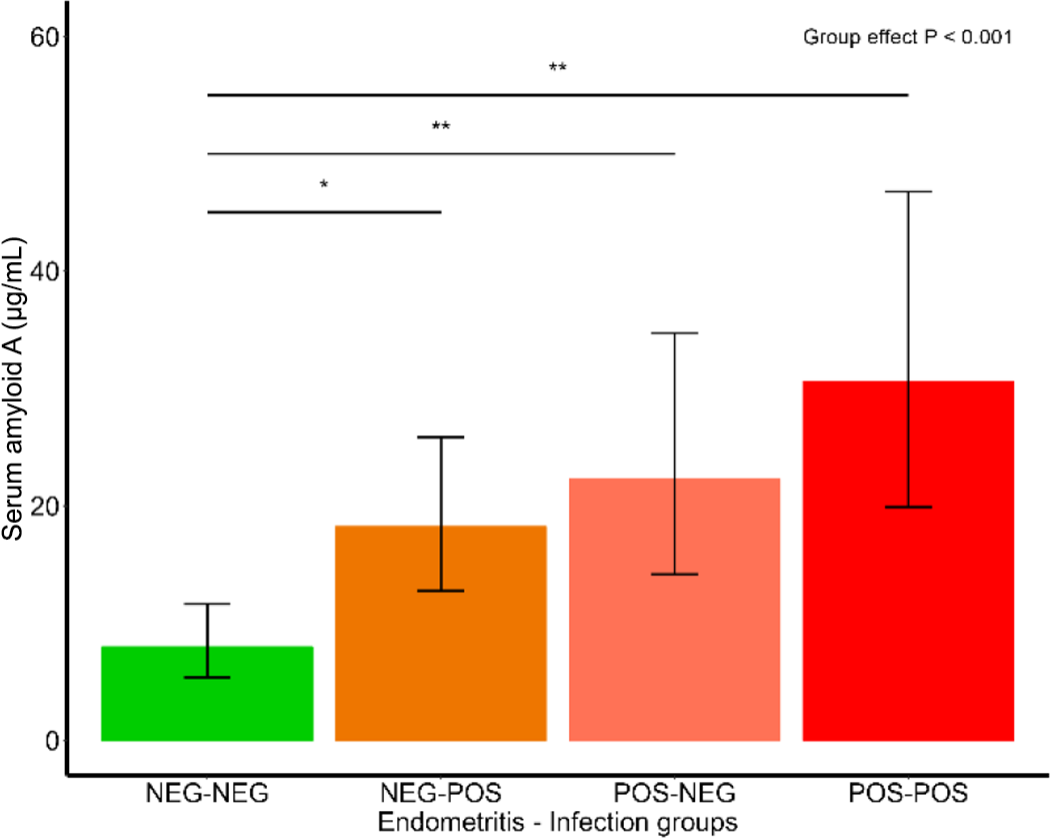
Geometric means and 95% LCI and UCI of concentrations of SAA for cows classified into uterine health groups of endometritis negative–infection negative (NEG–NEG), endometritis negative–infection positive (NEG–POS), endometritis positive–infection negative (POS–NEG), and endometritis positive–infection positive (POS–POS). * denotes pairwise comparison (*P* < 0.05), and ** denotes pairwise comparison (*P* < 0.01).

There is increasing evidence of the distinct pathogenesis of uterine diseases, including endometritis and purulent vaginal discharge. For example, one can be diagnosed in the absence of the other condition (Dubuc et al., 2010; Bogado Pascottini and LeBlanc, 2020), and diagnosis of either is associated with negative effects on conception and pregnancy outcomes (McDougall et al., 2009). Furthermore, when coincidental, they can be synergistic in their negative effects on reproduction (Dubuc et al., 2010). By inducing a uterine infection at 48 d postpartum in otherwise healthy cows, we aimed to provide a model of infection and the resultant acute inflammation without the homeorhetic changes associated with parturition and any associated metabolic and immune disturbances.

We hypothesized that circulating markers of inflammation would be increased in the BACT group; however, there was no difference for either SAA or α1-AGP concentrations between uterine infusion groups. The lack of difference between uterine infusion groups for SAA and α1-AGP concentrations appear contradictory to previous studies (Sheldon et al., 2001; Williams et al., 2005, 2007). In these studies, circulating concentrations of SAA and α1-AGP increased in the first 5 wk following calving, which was associated with reproductive tract inflammation and bacterial infection, including *T. pyogenes.* One reason for the apparent contradiction may lie in the experimental approaches. The previous studies used a naturally occurring peripartum infection model. In such a model, it is not possible to ascertain causation; the infection was associated with changes to the APR, characterized by increases in circulating inflammatory markers (Sheldon et al., 2001; Williams et al., 2005, 2007). However, it is unknown whether this is a coincidental effect of peripartum immune dysregulation, and the metabolic changes associated with the transition to lactation on the APR, or whether the homeorhetic changes associated with parturition contribute to the pathogenesis of uterine infection and postpartum diseases (Crookenden et al., 2016, 2019; LeBlanc, 2020).

Although there was an increase in uterine PMN in the days following infusion for the BACT group indicating a localized inflammatory reaction, some cows in the SAL group also exhibited an inflammatory response, with an increase in uterine PMN. It is possible that taking endometrial samples and the infusion of saline, or a combination of both, caused trauma to the endometrial lining, which triggered an inflammatory cascade and the influx of PMN. Cows were subsequently classified by the inflammatory response to the infusion, and the presence of *T. pyogenes* in bacterial culture. These 4 uterine health groups were used to compare concentrations of SAA and α1-AGP to investigate whether different responses to endometrial lining damage and bacterial ingress also had differences in the profile of inflammatory markers. Although the profile of inflammatory markers was not different between uterine health groups during the postinfusion period (i.e., a lack of group by time interaction), mean concentrations of SAA were greater in groups with an inflammatory response, or bacterial infection, or both, compared with cows that had no apparent inflammatory response to bacterial ingress or resolved the infection rapidly. Therefore, we hypothesize that there were differences in the immune response to the treatment protocol, which were reflected in the elevated concentrations of SAA in cows that had active inflammation or infection.

It is important to note the infection model in the current study was undertaken in healthy cows at d 48 postpartum, and the uterine environment may be different from that during the peripartum period. The uterine environment created by the process of the treatment protocol that introduces bacteria and trauma to the uterus was intended to be similar to that occurring during the parturition event, without the endocrine and metabolic signals which accompany parturition. By monitoring uterine PMN percentage and bacterial presence following bacterial infusion, the immune response to the challenge was characterized. The response appears to be different among individuals even without the additional metabolic and endocrine challenges of the periparturient period. It is likely that some individuals are predisposed to immune dysregulation, which may be exacerbated by additional challenges during the periparturient period, and whether the predisposition to immune dysfunction is under genetic control should be investigated.

Increased concentrations of inflammatory markers peripartum or in very early lactation could be indicative of a peripartum immune dysregulation that increases the risk of endometritis (Bogado Pascottini and LeBlanc, 2020; Spaans et al., 2023). Although there was no difference over the postinfusion period between uterine infusion groups, mean SAA concentrations for groups with uterine infection-induced endometritis, or bacterial infection with *T. pyogenes*, or both, were greater than cows with no inflammatory response to treatment protocols. Therefore, the degree and duration of the APR to uterine infection can be characterized by increased concentrations of circulating inflammatory markers, such as SAA.
